# Effects of ammonium-based ionic liquids and 2,4-dichlorophenol on the phospholipid fatty acid composition of zebrafish embryos

**DOI:** 10.1371/journal.pone.0190779

**Published:** 2018-01-17

**Authors:** Aleksandra Piotrowska, Anna Syguda, Bogdan Wyrwas, Lukasz Chrzanowski, Till Luckenbach, Hermann J. Heipieper

**Affiliations:** 1 Department of Environmental Biotechnology, Helmholtz Centre for Environmental Research-UFZ, Leipzig, Germany; 2 Faculty of Chemical Technology, Poznan University of Technology, Poznan, Poland; 3 Department of Bioanalytical Ecotoxicology, Helmholtz Centre for Environmental Research-UFZ, Leipzig, Germany; University of Louisville School of Medicine, UNITED STATES

## Abstract

Ionic liquids consisting of a combination of herbicidal anions with a quaternary ammonium cation act as efficient herbicides, which are under consideration to be used in the agriculture. In the present study, we used embryos of the zebrafish (*Danio rerio*) as a model to assess the toxic potential of ammonium-based ionic liquids for aquatic organisms. As we assumed interference of the partially hydrophobic ionic liquid cation with lipids, we investigated the adaptation response in the lipid composition of the zebrafish embryos, triggered by the ionic compound. Therefore, the impact of ammonium-based ionic liquids with different lengths of the alkyl chain ([C_6_,C_6_,C_1_,C_1_N][Br], [C_8_,C_8_,C_1_,C_1_N][Br]) on the phospholipid fatty acid (PLFA) profile of zebrafish embryos up to 72 hours post fertilization (hpf) was examined. Furthermore, the changes in the unsaturation index (UI) of PLFAs, as the sum parameter of membrane fluidity in eukaryotic cells, were presented. The PLFA’s UI in the zebrafish embryos upon exposure to quaternary ammonium salts was compared to the UI of the embryos upon exposure to nonionic 2,4-dichlorophenol, which has a similar hydrophobicity but is structurally different to [C_8_,C_8_,C_1_,C_1_N][Br]. It was shown that for ammonium-based ionic liquid precursors non-specific mode of action occurs and the toxic effect on lipid composition of zebrafish embryos can be well predicted based on chemical properties, like hydrophobicity. Furthermore, the changes in PLFAs, expressed by the UI, can be useful to study toxic effects of organic contamination. However, for zebrafish embryos, after ionic liquids and 2,4-DCP exposure, the changes were observed at high lethal concentrations, which caused the incidence of lethality of 30 and 50% of a group of test animals.

## Introduction

Ionic liquids (ILs) are considered as a ‘green’ alternative to many commonly used chemicals in industry and agriculture, such as 2,4-D, MCPA, MCPP and dicamba. The quaternary ammonium salt studied in this work, dimethyldioctylammonium bromide ([C_8_,C_8_,C_1_,C_1_N][Br]), contains a cation with long alkyl chains, which is used to synthetize herbicidal ionic liquids (HILs). The HIL, composed of a cation and an anion, has a melting point below the boiling point of water at atmospheric pressure, and in which at least one of the ions manifest herbicidal activity. The combination of a quaternary ammonium cation with an anion containing herbicide moieties (2,4-D, MCPA, MCPP, dicamba) allows to obtain herbicides demonstrating higher efficiency [1–4]. However, water soluble ionic liquid forms with long alkyl chains can influence the aquatic habitat. Especially, negative effects can be expected in large-scale applications in agriculture, where surface run-offs can lead to contamination of water bodies. The detection of IL in water is often limited by detection limits and the cost of analysis [5]. Therefore, there is a need to develop assays that could identify toxic effects of newly synthesized ionic compounds on aquatic organisms.

The effect of stressors on the lipid composition in cells or organisms has been studied before. It was found that generally the relative amounts of phospholipid saturated fatty acids is increased and that of unsaturated fatty acids is correspondingly decreased which enhances the lipid membrane rigidity [6–8]. As the membrane fluidity is increased by lipophilic compounds intercalating into the membrane the modification of the membrane composition can be seen as compensatory reaction [9]. Eukaryotic cells contain polyunsaturated fatty acids (PUFAs) [10] which regulate the cellular membranes`fluidity [11]. The adaptive response to a chemical stressor intercalating into the membrane becomes evident by changes in the phospholipid fatty acid (PLFA) composition and by the unsaturation index (UI) of the membrane. The UI is the sum parameter of membrane fluidity in eukaryotic cells and is expressed by the average number of double bonds in the fatty acid lipid fraction [12,13].

In this study, we investigated to which degree IL affects the lipid composition of zebrafish embryos that we chose as a model organism. The embryos of the zebrafish have become one of the most popular model systems in (eco)toxicology [14–18]. Furthermore, the earliest life-stages of animals are not defined as protected and do not fall into the regulation on animal experimentation. Therefore, zebrafish embryos are considered as replacement method for animal experiments [19]. Acute toxicity tests with zebrafish embryos (*Danio rerio*) show for many chemicals good correlations between results obtained with zebrafish embryo and acute adult fish toxicity tests [14,20]. Therefore, fish embryos can be regarded as alternative ecotoxicological model to adult fish [18]. Changes in the PLFA’s UI in zebrafish embryos treated with a toxic compound were first studied by Hachicho et al. (2015) [21], who examined the effect of the moderately lipophilic compound 2,4-dinitrophenol (2,4-DNP; log P = 1.67; ChemIDplus a TOXNET database) at LC_10_ (concentration lethal for 10% of experimental organisms). 2,4-DNP caused a by 7 to 23% significant decrease in the PLFA’s UI in zebrafish embryos [21].

In this study we addressed the question how the ionic liquid precursor [C_8_,C_8_,C_1_,C_1_N][Br] as a charged compound affects the lipid composition in the zebrafish embryos. Furthermore, the unsaturation index (UI) of phospholipid fatty acids (PLFAs) was determined as a measure for membrane rigidity. The differences in chemical behavior of ionic and nonionic compounds can lead to differences e.g. in sorption to membranes [22], which may therefore lead to differences in effects to the lipid composition. As a nonionic reference compound with a similar log P as [C_8_,C_8_,C_1_,C_1_N][Br] served 2,4-dichlorophenol (2,4-DCP), which exists in both the non-dissociated and charged form in environmental soil and water (ChemIDplus a TOXNET database). Furthermore, [C_8_,C_8_,C_1_,C_1_N][Br] effects were compared to those of the structurally similar but less hydrophobic an ammonium-based ionic liquid precursor with shorter alkyl chains ([C_6_,C_6_,C_1_,C_1_N][Br]).

## Materials and methods

### Quaternary ammonium salts

Dihexyldimethylammonium bromide ([C_6_,C_6_,C_1_,C_1_N][Br]) and dimethyldioctylammonium bromide ([C_8_,C_8_,C_1_,C_1_N][Br]) were designed and synthetized in the Department of Chemical Technology in Poznan University of Technology, Poland. Synthesis and physico-chemical properties are provided in S1 Appendix. All studied quaternary ammonium salts are water-soluble [23].

### Collection of eggs and cultivation of embryos

The study was performed with eggs/embryos of the zebrafish, *Danio rerio* (Hamilton- Buchanan, 1822). Adult zebrafish were obtained from a local supplier and were maintained and bred according to standard protocols [24]. Collection of eggs and cultivation of embryos were performed as described in [16].

### Fish embryo acute toxicity test

Fish embryo acute toxicity (FET) tests were carried out as described in [25]. Fertilized zebrafish eggs were exposed to different concentrations of test chemicals, [C_6_,C_6_,C_1_,C_1_N][Br], [C_8_,C_8_,C_1_,C_1_N][Br] and 2,4-DCP (Sigma Aldrich, Munich, Germany) for 48 h. After 24 h and 48 h, four observations were noted as indicators of lethality: (i) coagulation of fertilized eggs, (ii) lack of somite formation, (iii) lack of detachment of the tail-bud from the yolk sac, and (iv) lack of heartbeat. At the end of the exposure period, acute toxicity was determined based on a positive outcome in any of the four apical observations recorded. The lethal concentrations were interpolated from obtained dose-response curves.

### Exposure experiments

[C_8_,C_8_,C_1_,C_1_N][Br], [C_6_,C_6_,C_1_,C_1_N][Br] and 2,4-DCP were dissolved in the embryo culture water according to [25]. The concentrations of compounds in exposure medium (the embryo culture water with toxic compound) corresponded to the interpolated lethal concentrations from FET tests. Freshly fertilized zebrafish eggs were added to 0.7 mL of exposure medium per egg and incubated at 27°C ±1 for 0.5 h, 24 h, 48 h and 72 h. For control, freshly fertilized zebrafish eggs were added to 0.7 mL of exposure medium without toxic compounds and incubated at 27°C ± 1 for 0.5 h, 24 h, 48 h and 72 h. The exposure experiments were initiated as soon as possible after fertilization of the eggs. Pools of 50 surviving individuals after 0.5, 24, 48 and 72 h were frozen at -80°C in 1.5 mL Eppendorf reaction tubes and stored for a maximum of one week prior to extraction.

### Lipid extraction

Lipid extraction from zebrafish embryos was carried out according to Hachicho et al. (2015) [21], which is the modification of a method described by Bligh and Dyer (1959) [26]. Frozen embryos were suspended in 1 mL potassium phosphate buffer (0.05 M, pH 7) and transferred to a FastPrep tube (MP Biomedicals, Lysin Matrix E, 2 mL, Ref. 6914–100). For tissue homogenization, the FastPrep mill (MP Biomedicals, FastPrep-24) was used at speed 6 m/s for 30 sec. The content of each FastPrep tube was transferred to a 15 mL glass vial with 1 mL potassium phosphate buffer. Each FastPrep tube was repeatedly washed with 4 × 1 mL methanol, which was transferred to the 15 mL glass vial. One additional mL methanol and 2.5 mL chloroform were added to the suspension, which was shaken for 2 h at 350 rpm on an orbital shaker at room temperature. Subsequently, 2.5 mL deionized water and 2.5 mL chloroform were added and samples were left at 5°C for 18 h for phase separation. The chloroform phase was transferred to a clean glass vial and the solvent was evaporated under a stream of nitrogen. The dried components were dissolved in 0.2 mL chloroform.

### Separation and derivatization of phospholipids

Separation of different lipid classes was performed by passage of the samples over approximately 0.5 g silica gel (Unisil activated silicic acid; Clarkson Chromatography Products Inc., SouthWilliamsport, PA, USA) in a glass column (BAKERBOND SPE; 8 mL; J.T.Baker, Deventer, Netherlands) containing a PTFE disc. Prior to addition of the sample, the packed column was conditioned by successively washing with 5 mL ammonium acetate (0.02 M in methanol), 5 mL acetone and 5 mL chloroform. Neutral lipids were eluted from the silica gel with 10 mL chloroform, glycolipids with 10 mL acetone and phospholipids with 10 mL methanol. The fraction of phospholipids was collected separately in glass vials and dried under a gentle stream of nitrogen gas.

The fatty acids in the phospholipid fraction were derivatized to fatty acid methyl esters (FAME) as described by Morrison and Smith (1964) [27]. Methylation was carried out with 1.2 mL boron trifluoride methanol complex (20% in methanol; Sigma Aldrich, Munich, Germany) at 95°C for 15 min. The reaction was stopped by adding 0.6 mL deionized water and 1 mL hexane (HPLC grade; Sigma Aldrich, Munich, Germany) After 60 sec shaking (Vortex-Genie 2 Shaker), an aliquot of 0.5 mL was taken from the hexane phase of each sample. The solvent was evaporated under a stream of nitrogen and the dried sample was dissolved in 0.5 mL n-hexane including internal standard (10 μg/mL methyl heneisosanoate [C21:0]; Sigma Aldrich, Munich, Germany). Samples were stored at 5°C until analysis by gas chromatography/mass spectrometry (GC/MS) was performed.

### GC-MS analysis and determination of the unsaturation index (UI)

FAME samples were analyzed by GC/MS using a gas chromatograph (7890A GC system, Agilent Technologies, Waldbronn, Germany) coupled to a mass spectrometer (MS, 5975C inert MSD with triple axis detector, Agilent Technologies) and equipped with an autosampler (7693, Agilent Technologies) and a split/split-less injector (G4513A, Agilent Technologies). The column was a BPX5 capillary column (30 m × 0.25mm × 0.25 μm; SGE, Kiln Farm Milton Keynes, UK), and helium at a flow rate of 1.2 mL min^-1^ was used as carrier gas. The injection volume was 1 μl. The injection was splitless, the injector temperature was 280°C. The initial oven temperature was 50°C for 1 min and the final temperature was 300°C with an increase of 4°C min^-1^ to 250°C and 20°C min^-1^ to 300°C followed by 5 min isothermal conditions. The temperature of the transfer line to the MS was set to 300°C. Ionization of the eluting compounds was performed by electron impact at 70 eV. The ion source temperature was 230°C and the quadrupole temperature was 150°C. Full scans were acquired from m/z 40 to m/z 500. Data acquisition was performed using the MSD-Chemstation Software (MSD ChemStation E.02.00.493; Agilent Technologies). Fatty acids were identified by comparison of the retention times of the peaks with those of a commercial qualitative standard of bacterial acid methyl esters (BAME, Sigma Aldrich) and of the mass spectra with the NIST data base using the NIST Mass Spectral Search Programm (NIST MS Search 2.0f). The internal standard of known concentration allowed to validate the method.

The relative amounts of the individual fatty acids were used to calculate the unsaturation index (UI) [12,13,21]:

UI=[(%16:1+%18:1)+(%18:2x2)+(%18:3x3)+…]/100

### Statistical analysis

All the experiments were carried out in triplicates. Mean values and standard deviations were determined from replicate values. For determining statistically significant changes of the UI values, the ANOVA followed by Dunnett’s test with 0.5 hpf as control group was performed. Student’s t-test was applied to compare the differences in phospholipid patterns for treatments (LC_50_) with untreated embryos (control). For [C_8_,C_8_,C_1_,C_1_N][Br] where more than two groups were compered (control, LC_10_, LC_30_, LC_50_) the ANOVA followed by Dunnett’s test was performed. Differences were regarded significant if P < 0.05.

## Results

### Acute toxicity studies

The acute toxicities of [C_8_,C_8_,C_1_,C_1_N][Br], [C_6_,C_6_,C_1_,C_1_N][Br] and 2,4-DCP to zebrafish embryos were estimated. The dose-response curves and outcomes are given in S1 Fig and S1 Table. The chosen lethal concentrations for each compound are shown in Table 1. The purpose of the tests was to define lethal concentrations for exposure experiments and phospholipid analysis.

**Table 1:** Summary of acute toxicity data for each compound.

Compound	LC_10_ [mM]	LC_30_ [mM]	LC_50_ [mM]
[C_8_,C_8_,C_1_,C_1_N][Br]	0.093	0.123	0.147
[C_6_,C_6_,C_1_,C_1_N][Br]	-	-	3.423
2,4-DCP	-	-	0.026

LC_10_, LC_30_, LC_50_, concentrations of the compound which cause the incidence of lethality of 10%, 30% and 50% of a group of test animal. (-): not interpolated.

### Changes in the phospholipid fatty acid pattern after chemical exposure

**Fig 1:**
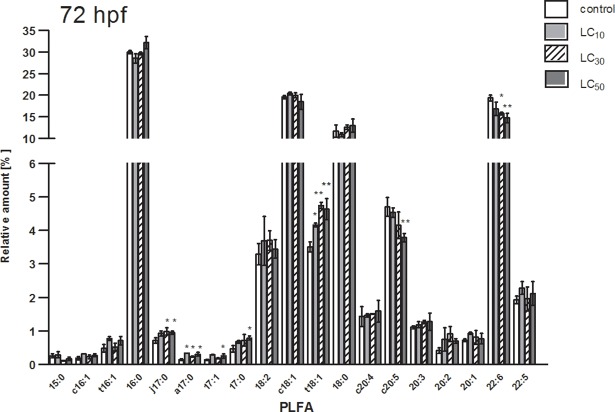
PLFA pattern of untreated zebrafish embryos (control) and embryos treated with [C_8_,C_8_,C_1_,C_1_N][Br] (LC_10_, LC_30_, LC_50_) at 72 hpf. hpf: hours post fertilization; y-axis is divided into two sections with different scales; *: P < 0.05; **: P < 0.01.

The differences in phospholipid patterns for [C_8_,C_8_,C_1_,C_1_N][Br] treated (LC_10_, LC_30_, LC_50_) and untreated embryos (control) after the longest exposure time (72 hpf) are presented in Fig 1. For polyunsaturated docosahexaenoic acid (C22:6) the relative amount was statistically lower than for the control, where changes could be observed for LC_50_ as well as for LC_30_. In a case of other PLFAs, significantly higher contents were observed for Cj17:0, Ca17:0, C17:0, C17;1, Ct18:1. For eicosapentaenoic acid (Cc20:5) relative abundance was lower, compared to control for LC_50_. No statistically significant differences were observed for LC_10_ (Fig 1). The differences in phospholipid patterns for [C_8_,C_8_,C_1_,C_1_N][Br] treated (LC_10_, LC_30_, LC_50_) and untreated embryos at 0.5, 24 and 48 hpf are provided in S2–S4 Figs.

### Effect of toxic exposure on UI values

**Fig 2:**
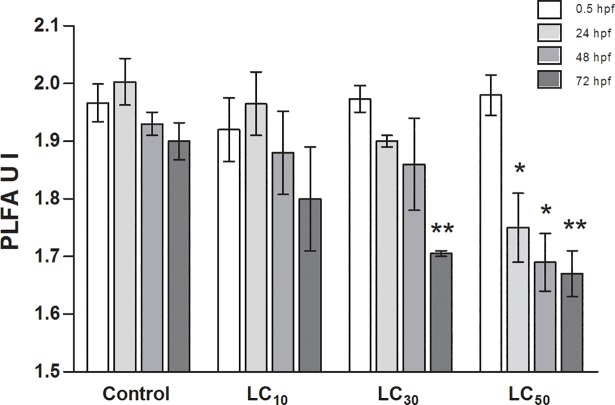
Mean values (±STD) for unsaturation index (UI) of the PLFA fraction in untreated zebrafish embryos (control) and embryos treated with [C_8_,C_8_,C_1_,C_1_N][Br] (LC_10_, LC_30,_ LC_50_) at 0.5, 24, 48 and 72 hpf. hpf: hours post fertilization; *: P < 0.05; **: P < 0.01.

Based on the relative abundance of PLFAs the unsaturation index (UI) was calculated as sum parameter of membrane fluidity in eukaryotic cells. The differences in the UI values of [C_8_,C_8_,C_1_,C_1_N][Br] treated (LC_10_, LC_30_, LC_50_) and untreated embryos at 0.5, 24, 48 and 72 hpf are presented in Fig 2. The UI values for untreated embryos were between 1.9 and 2.0. For treated embryos with [C_8_,C_8_,C_1_,C_1_N][Br] the UI values were statistically significantly lower for LC_50_, respectively at 24, 48 and 72 hpf. Additionally, the UI value for treated embryos at 72 hpf for LC_30_ was significantly lower than in untreated embryos. No statistically significant differences were observed for LC_10_ (Fig 2).

### Influence of different chemical structures on UI values

**Fig 3:**
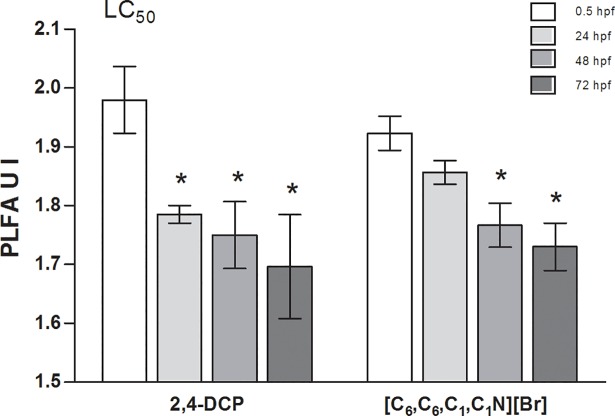
Mean values (±STD) for unsaturation index (UI) of the PLFA fraction in zebrafish embryos treated with 2,4-DCP and [C_6_,C_6_,C_1_,C_1_N][Br] for LC_50_ at 0.5, 24, 48 and 72 hpf. hpf: hours post fertilization; *: P < 0.05; **: P < 0.01.

The difference in the UI values of 2,4-DCP and [C_6_,C_6_,C_1_,C_1_N][Br] treated zebrafish embryos for LC_50_ at 0.5, 24, 48 and 72 hpf was presented in Fig 3. For treated embryos with 2,4-DCP the UI values were statistically significantly lower at 24, 48 and 72 hpf as compared to 0.5 hpf. For [C_6_,C_6_,C_1_,C_1_N][Br] a statistically significant difference in the UI values between treated zebrafish embryos at 0.5 hpf and treated embryos at 48 and 72 hpf was observed (Fig 3). At 48 and 72 hpf the UI values for zebrafish embryos treated with [C_8_,C_8_,C_1_,C_1_N][Br], 2,4-DCP and [C_6_,C_6_,C_1_,C_1_N][Br] were comparable, and approximately 1.7–1.8. The differences in phospholipid fatty acid patterns for 2,4-DCP and [C_6_,C_6_,C_1_,C_1_N][Br] treated (LC_50_) and untreated embryos up to 72 hpf are provided in S5–S12 Figs.

## Discussion

In this study we addressed the question how a ionic liquid as a charged compound affects the lipid composition in the zebrafish embryos and whether differences in chemical behavior of ionic and nonionic compounds lead to differences in effects on the lipid composition. The ability of a chemical compound to interact with cellular membranes, causing disruption and increasing its fluidity, can activate an adaptation response. This adaptive process is called ‘homeoviscous adaptation’ [28] and is based on the balance between saturated and unsaturated fatty acids. If a chemical intercalates in the membrane affecting its fluidity the relative amount of saturated fatty acids increases and, correspondingly, unsaturated fatty acids decrease resulting in a decrease of the fluidity of the cellular membrane counteracting the fluidizing effect of the chemical compound [11,28]. The changes in fatty acid pattern of the phospholipid fraction indicate that the ammonium-based ionic liquid precursor ([C_8_,C_8_,C_1_,C_1_N][Br]) interacts with cellular membranes and triggers an adaptation mechanism. Therefore, [C_8_,C_8_,C_1_,C_1_N][Br] and 2,4-DCP have comparable hydrophobicity and for them similar decrease in the UI at 24, 48 and 72 hpf was observed. However, the fact that 2,4-DCP was more toxic for zebrafish embryos than [C_8_,C_8_,C_1_,C_1_N][Br] was not pronounced in the UI. As a second compound to observe the influence of different chemical structure on the UI values, the more hydrophilic ammonium-based ionic liquid precursor ([C_6_,C_6_,C_1_,C_1_N][Br]) with the log P = 1.08 [23] was chosen. For [C_6_,C_6_,C_1_,C_1_N][Br] the statistically significant decrease in the UI was observed later at 48 hpf. This indicates that the more hydrophobic compound causes higher membrane disruption and triggers a stronger adaptation response which gives significant results in the UI. Furthermore, it was shown that for ammonium-based ionic liquid precursors non-specific mode of action occurs and the toxic effect can be well predicted by models for ‘baseline toxicity’ also called narcosis [29]. The mechanism of narcosis is based on partitioning of a toxic compound into membranes, leading to its non-specific reversible disturbance and increase in the fluidity [30]. It was deduced, that the mortality of the substances only occurs when the membrane fluidity is affected. Therefore, the next step on further investigation of the influence of toxic compounds on changes in the PLFA pattern would be the presentation of correlation between LC values with the effect on the UI values. In conclusion, studying the changes in PLFAs, expressed by the UI, can be useful to measure toxic effects of organic contamination. However, for zebrafish embryos, after ionic liquids and 2,4-DCP exposure, the changes were observed at high lethal concentrations, which caused the incidence of lethality of 30 and 50% of a group of test animal.

## Ethics statement

All zebrafish husbandry and experimental procedures were performed in accordance with the German animal protection standards and were approved by the Government of Saxony, Landesdirektion Leipzig, Germany (Aktenzeichen 75–9185.64) and were based on the guidelines on the protection of experimental animals by the Council of Europe, Directive 2010-63-EU, which allows zebrafish embryos to be used up to the moment of independent feeding (approximately 5 days after fertilization). The embryos used here in experiments were not older than 4 days and therefore no authorization was required by the local authority.

## Supporting information

S1 Appendix Synthesis of dialkyldimethylammonium bromides. Quantitative analysis, preparation and physico-chemical properties of dialkyldimethylammonium bromides.
(PDF)

S1 Table Interpolated concentrations and given parameters for dose-response curves. LC_10_, LC_30_, LC_50_, concentrations of the compound which cause consequently the incidence of lethality of 10%, 30% and 50% of a group of test animal; (CI) confidence interval; (-): not interpolated/calculated.
(TIF)

S1 Fig Dose-response curves. Dose-response curve in mortality [%] and concentrations [mM] of 2,4-DCP, [C_8_,C_8_,C_1_,C_1_N][Br] and [C_6_,C_6_,C_1_,C_1_N][Br] respectively of zebrafish embryos.
(TIF)

S2 Fig PLFA pattern of untreated zebrafish embryos (control) and embryos treated with [C_8_,C_8_,C_1_,C_1_N][Br] (LC_10_, LC_30_, LC_50_) at 0.5 hpf. hpf: hours post fertilization; y-axis is divided into two sections with different scales; *: P < 0.05; **: P < 0.01.
(TIF)

S3 Fig PLFA pattern of untreated zebrafish embryos (control) and embryos treated with [C_8_,C_8_,C_1_,C_1_N][Br] (LC_10_, LC_30_, LC_50_) at 24 hpf. hpf: hours post fertilization; y-axis is divided into two sections with different scales; *: P < 0.05; **: P < 0.01.
(TIF)

S4 Fig PLFA pattern of untreated zebrafish embryos (control) and embryos treated with [C_8_,C_8_,C_1_,C_1_N][Br] (LC_10_, LC_30_, LC_50_) at 48 hpf. hpf: hours post fertilization; y-axis is divided into two sections with different scales; *: P < 0.05; **: P < 0.01.
(TIF)

S5 Fig PLFA pattern of untreated zebrafish embryos (control) and embryos treated with 2,4-DCP (LC_50_) at 0.5 hpf. hpf: hours post fertilization; y-axis is divided into two sections with different scales; *: P < 0.05; **: P < 0.01.
(TIF)

S6 Fig PLFA pattern of untreated zebrafish embryos (control) and embryos treated with 2,4-DCP (LC_50_) at 24 hpf. hpf: hours post fertilization; y-axis is divided into two sections with different scales; *: P < 0.05; **: P < 0.01.
(TIF)

S7 Fig PLFA pattern of untreated zebrafish embryos (control) and embryos treated with 2,4-DCP (LC_50_) at 48 hpf. hpf: hours post fertilization; y-axis is divided into two sections with different scales; *: P < 0.05; **: P < 0.01.
(TIF)

S8 Fig PLFA pattern of untreated zebrafish embryos (control) and embryos treated with 2,4-DCP (LC_50_) at 72 hpf. hpf: hours post fertilization; y-axis is divided into two sections with different scales; *: P < 0.05; **: P < 0.01.
(TIF)

S9 Fig PLFA pattern of untreated zebrafish embryos (control) and embryos treated with [C_6_,C_6_,C_1_,C_1_N][Br] (LC_50_) at 0.5 hpf. hpf: hours post fertilization; y-axis is divided into two sections with different scales; *: P < 0.05; **: P < 0.01.
(TIF)

S10 Fig PLFA pattern of untreated zebrafish embryos (control) and embryos treated with [C_6_,C_6_,C_1_,C_1_N][Br] (LC_50_) at 24 hpf. hpf: hours post fertilization; y-axis is divided into two sections with different scales; *: P < 0.05; **: P < 0.01.
(TIF)

S11 Fig PLFA pattern of untreated zebrafish embryos (control) and embryos treated with [C_6_,C_6_,C_1_,C_1_N][Br] (LC_50_) at 48 hpf. hpf: hours post fertilization; y-axis is divided into two sections with different scales; *: P < 0.05; **: P < 0.01.
(TIF)

S12 Fig PLFA pattern of untreated zebrafish embryos (control) and embryos treated with [C_6_,C_6_,C_1_,C_1_N][Br] (LC_50_) at 72 hpf. hpf: hours post fertilization; y-axis is divided into two sections with different scales; *: P < 0.05; **: P < 0.01.
(TIF)
