# Supplementary material for: Effects of ammonium-based ionic liquids and 2,4-dichlorophenol on the phospholipid fatty acid composition of zebrafish embryos
Source: PLoS One. 2018 Jan 17;13(1):e0190779. doi: 10.1371/journal.pone.0190779 (PMC5771613; doi:10.1371/journal.pone.0190779)
Supplement: S1 Appendix — Quantitative analysis, preparation and physico-chemical properties of dialkyldimethylammonium bromides. (PDF) [file pone.0190779.s001.pdf]

Synthesis of dialkyldimethylammonium bromides

Chemical reagents

The starting materials and reagents used in preparing the disclosed compounds were purchased from Sigma-Aldrich (Munich, Germany): hexyldimethylamine (98%), dimethyloctylamine (95%), 1-bromohexane (98%), 1-bromooctane (99%), and from Avantor: hexane (99%), acetone (99%). Reagents for titration were purchased from Aldrich: sodium tetraphenylborate (99.5%) and from Avantor: methanol (99.8%), chloroform (98%), sodium hydroxide (98%) and bromophenol blue (100%).

NMR Spectroscopy

^{1}H NMR spectra were recorded on a Varian VNMR-S spectrometer operating at 400 MHz with tetramethylsilane as the internal standard. ^{13}C NMR spectra were obtained with the same instrument at 100 MHz. CHN elemental analyses were performed at Adam Mickiewicz University, Poznan.

Quantitative analysis of the quaternary ammonium bromides

The concentration of dialkyldimethylammonium bromides (purity) was determined according to the extractive titration method [1,2] in alkaline water-chloroform system using sodium tetraphenylborate as the titrant and the bromophenol blue in the acid form (0.2% in methanol) as the indicator. The indicator is soluble in water and insoluble in chloroform, but can be extracted into chloroform as blue ion complex with dialkyldimethylammoniumcation. When the dialkyldimethylammonium salt as the complex with bromophenol blue is titrated with sodium tetraphenylborate solution, the dialkyldimethylammonium tetraphenylborate is formed. Exchanged bromophenol blue anion with sodium cation it is not soluble in

chloroform and turns on to the water phase to give purple color. The end point is marked by the appearance of purple color of indicator in alkaline aqueous layer after decomposition of the chloroform-soluble blue complex and then the chloroform layer turns from blue to colorless. In a Erlenmeyer flask with a glass stopper 0.1 ± 0.0001 g of the sample was placed and blended with 30 mL of chloroform, 10 mL of NaOH water solution (1 mol/L) and six drops of indicator. The resulting biphasic system was titrated with 0.01 mol/L of sodium tetraphenylborate solution. After addition of a titrant increment, the flask was stopped and the sample was stirred vigorously for is essential to avoid over titration.

The purity as the concentration of dialkyldimethylammonium bromides was calculated using equation:

$$X=\frac{C_{TFB}\cdot V_{TFB}\cdot M}{1000\cdot m_s}\cdot 100\ [\%]$$

where:

X – concentration of dialkyldimethylammonium bromides [%]

C_{TFB} – concentration of sodium tetraphenylborate [mol/L]

VTFB – volume of the titrant solution [mL]

M – molecular weight of analyzed compound [g/mol]

m_s – mass of the analyzed sample [g]

Preparation of dialkyldimethylammonium bromides

0.1 mol of 1-bromoalkane (1-bromohexane or 1-bromooctane) was added into a round-bottomed flask which contained solution of 0.1 mol adequate alkyldimethylamine (hexyldimethylamine or dimethyloctylamine) in 20 mL acetone. The reaction mixture was vigorously stirred at room temperature for 24 h. Afterwards acetone was evaporated and crude

product was cooled in the freezer for 24 hours. The precipitate was filtered, washed with cold hexane and dried at 50 °C under reduced pressure (13 hPa).

Dihexyldimethylammonium bromide ([C_6,C_6,C_1,C_1N][Br]) ^{1}H NMR ($CDCl_3$) δ ppm = 0.86 (t, J = 7.2 Hz, 6H), 1.30 (m, 12H), 1.68 (q, J = 7.2 Hz, 4H), 3.37 (s, 6H), 3.50 (t, J = 8.6 Hz, 4H); ^{13}C NMR δ ppm = 13.4, 21.9, 22.2, 25.4, 30.7, 50.8, 63.4. Anal. Calcd for $C_{14}H_{32}NBr$: C 57.13, H 10.96, N 4.69; Found: C 56.76, H 11.17, N 4.24.

Dimethyldioctylammonium bromide ([C_8,C_8,C_1,C_1N][Br]) ^{1}H NMR ($CDCl_3$) δ ppm = 0.80 (t, J = 7.0 Hz, 6H), 1.20 (m, 20H), 1.64 (q, J = 7.9 Hz, 4H), 3.31 (s, 6H), 3.45 (t, J = 8.6 Hz, 4H); ^{13}C NMR δ ppm = 13.5, 22.0, 22.2, 25.7, 28.5, 28.6, 31.1, 50.7, 63.3. Anal. Calcd for $C_{18}H_{40}NBr$: C 61.68, H 11.53, N 4.00; Found: C 61.29, H 11.32, N 3.91.

Properties of dialkyldimethylammonium bromides

[C_6,C_6,C_1,C_1N][Br] at 25 °C is hygroscopic microcrystalline gel, purity 97%, yield 87%. [C_8,C_8,C_1,C_1N][Br] at 25 °C is hygroscopic solid (melting point 81-86°C), purity 98.5%, yield 91%.

References

1. Cross JT. The identification and determination of cationic surface-active agents with sodium tetraphenylboron, Analyst. 1965;90: 315-324.
2. Wang D-H, Weng H-S. Solvent and salt effects on the formation of third liquid phase and the reaction mechanisms in the phase transfer catalysis system-reaction between n-butyl bromide and sodium phenolate, Chem. Eng. Sci. 1995;50: 3477-3486.
