# Supplementary figures and images for: Effects of ammonium-based ionic liquids and 2,4-dichlorophenol on the phospholipid fatty acid composition of zebrafish embryos
Source: PLoS One. 2018 Jan 17;13(1):e0190779. doi: 10.1371/journal.pone.0190779 (PMC5771613; doi:10.1371/journal.pone.0190779)

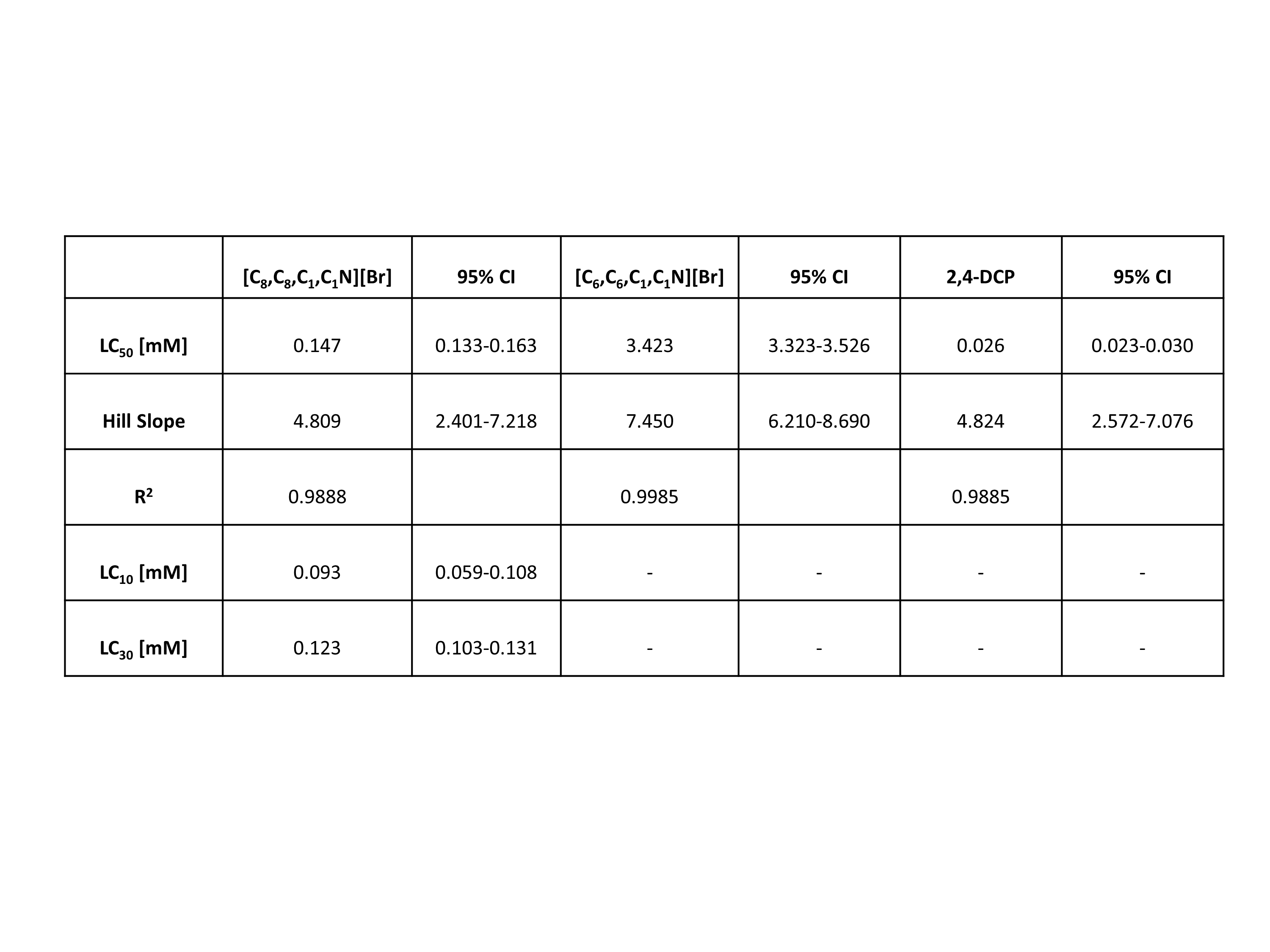

Supplement: S1 Table — LC10, LC30, LC50, concentrations of the compound which cause consequently the incidence of lethality of 10%, 30% and 50% of a group of test animal; (CI) confidence interval; (-): not interpolated/calculated. (TIF) [file pone.0190779.s002.tif]

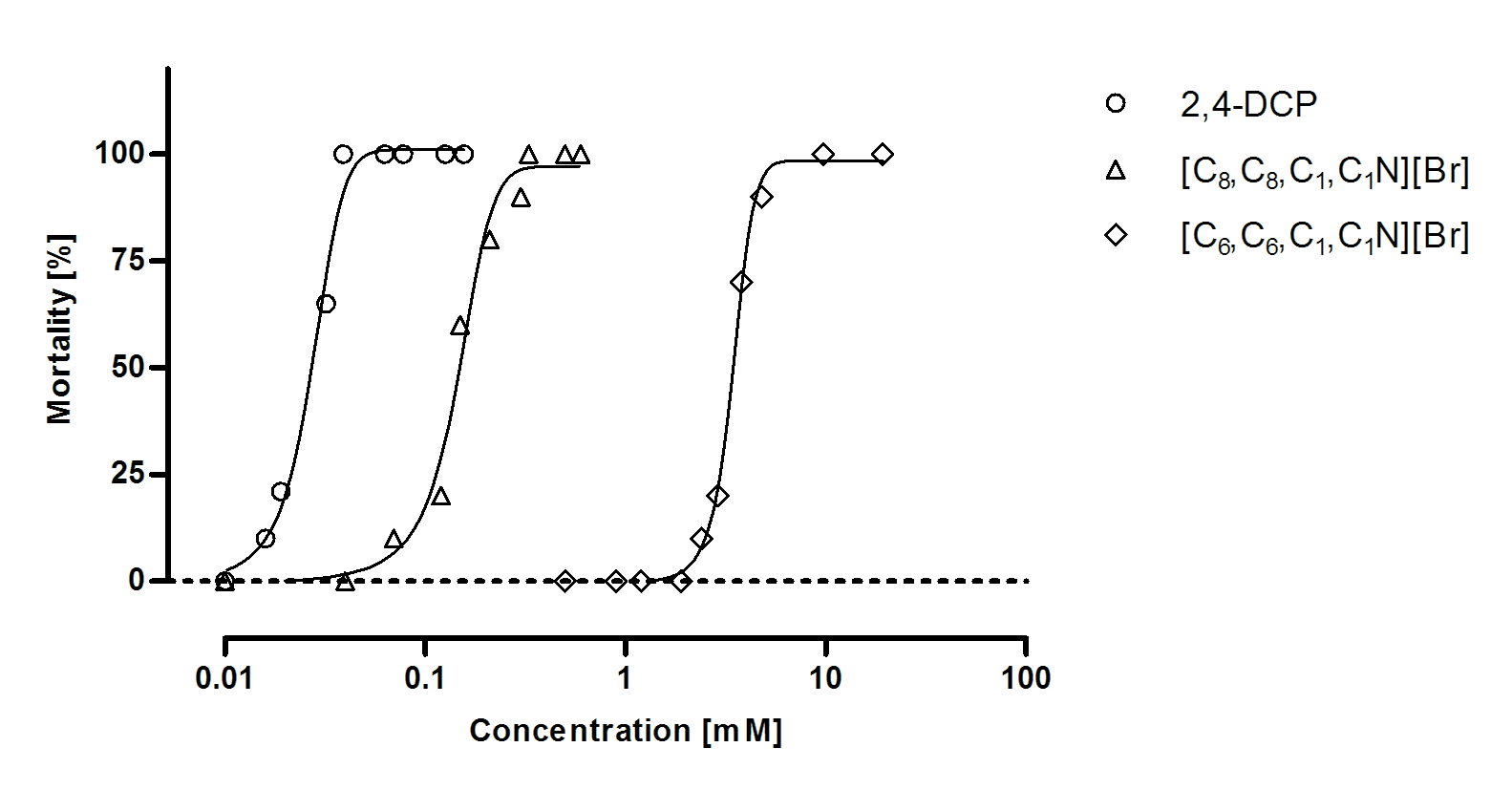

Supplement: S1 Fig — Dose-response curve in mortality [%] and concentrations [mM] of 2,4-DCP, [C8,C8,C1,C1N][Br] and [C6,C6,C1,C1N][Br] respectively of zebrafish embryos. (TIF) [file pone.0190779.s003.tif]

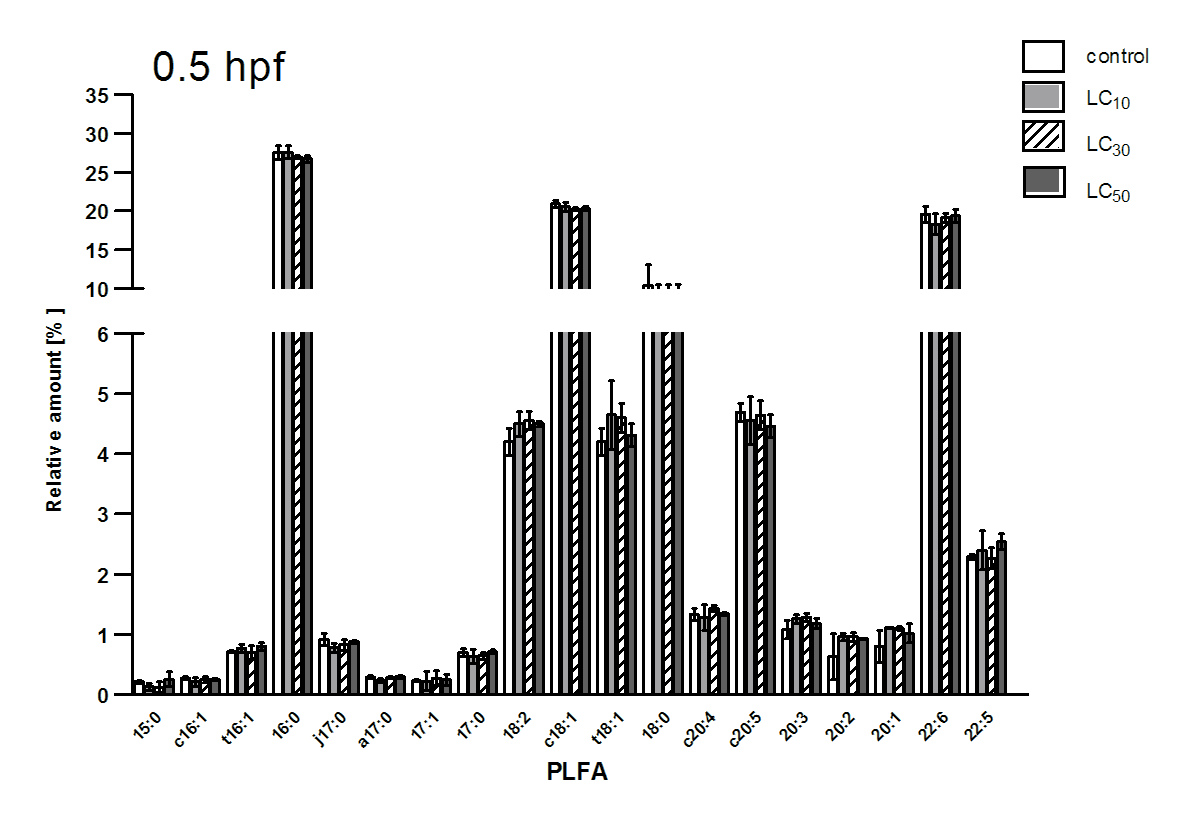

Supplement: S2 Fig — hpf: hours post fertilization; y-axis is divided into two sections with different scales; *: P < 0.05; **: P < 0.01. (TIF) [file pone.0190779.s004.tif]

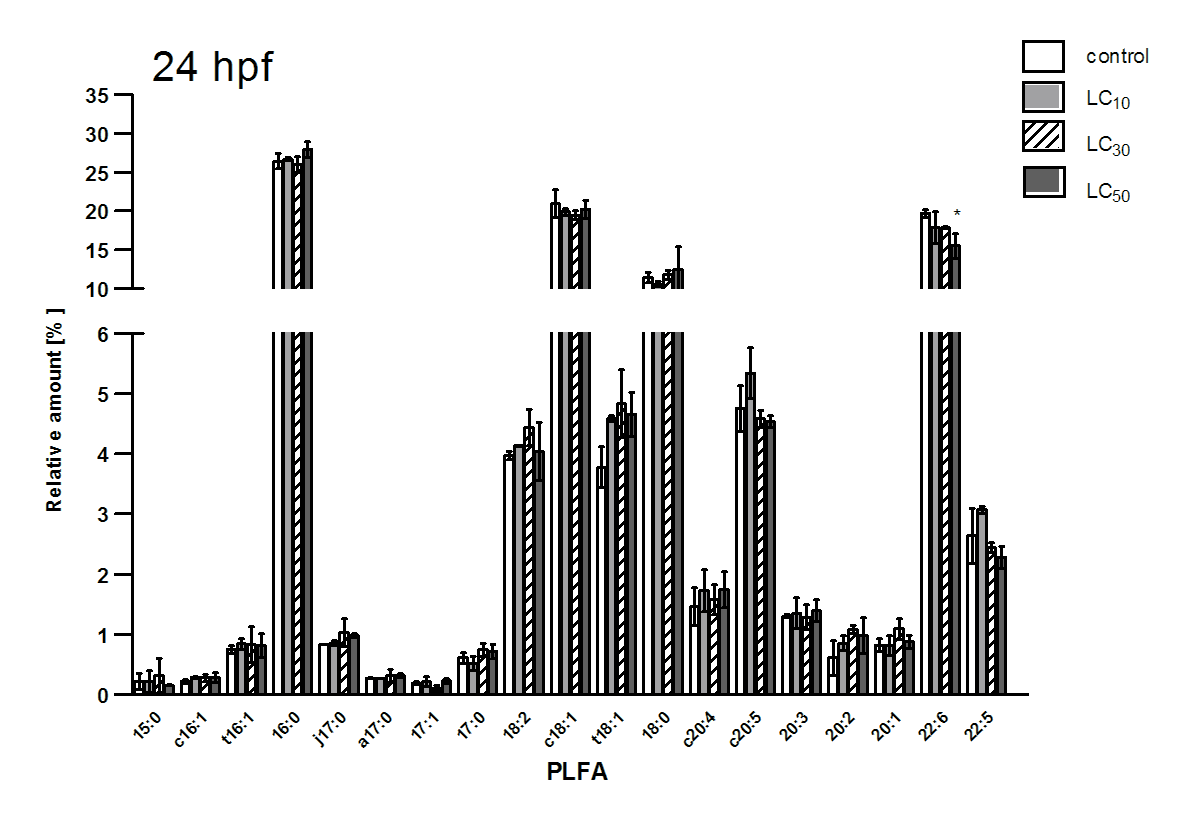

Supplement: S3 Fig — hpf: hours post fertilization; y-axis is divided into two sections with different scales; *: P < 0.05; **: P < 0.01. (TIF) [file pone.0190779.s005.tif]

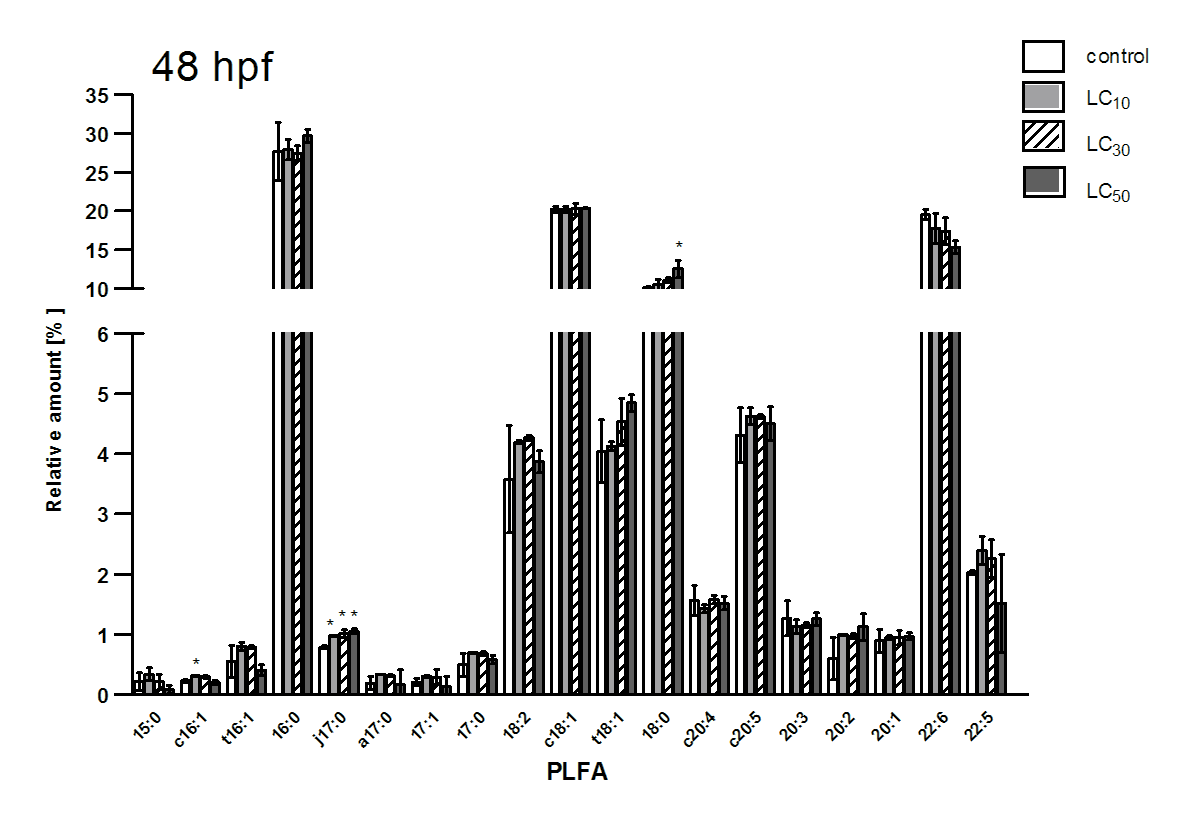

Supplement: S4 Fig — hpf: hours post fertilization; y-axis is divided into two sections with different scales; *: P < 0.05; **: P < 0.01. (TIF) [file pone.0190779.s006.tif]

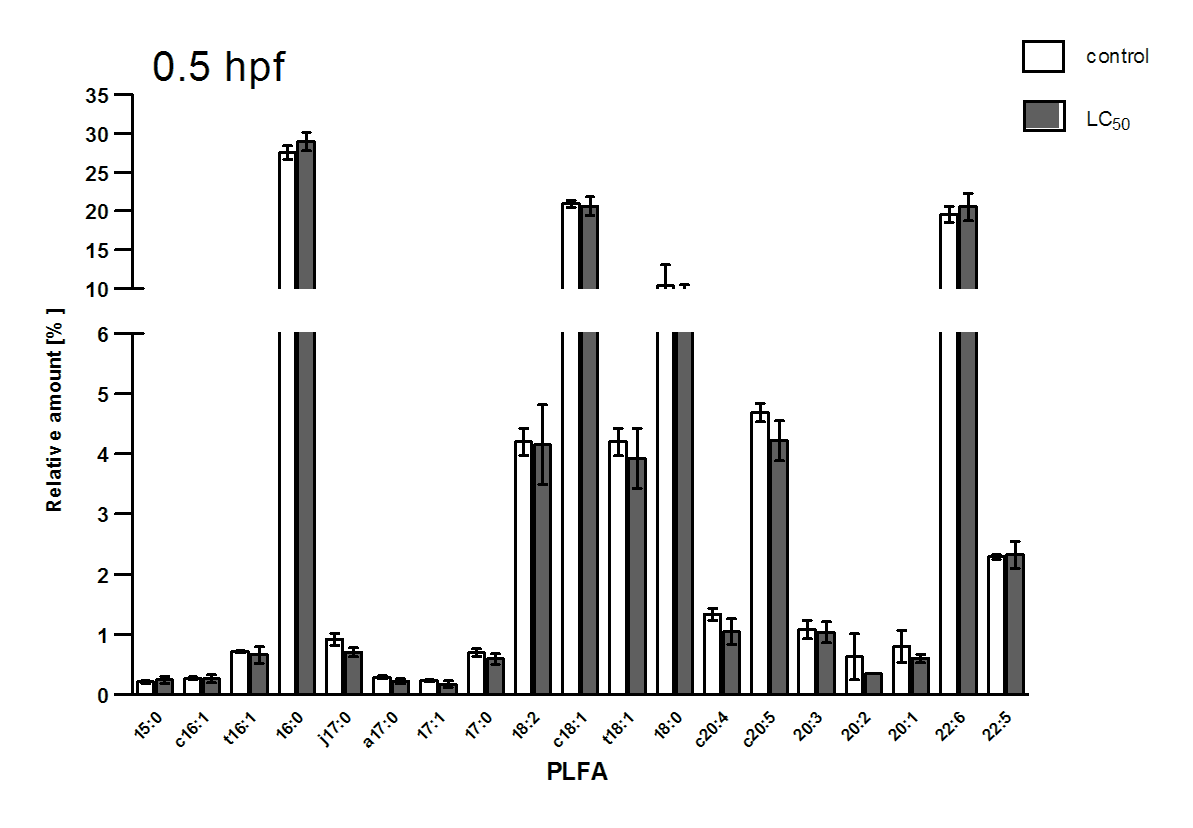

Supplement: S5 Fig — hpf: hours post fertilization; y-axis is divided into two sections with different scales; *: P < 0.05; **: P < 0.01. (TIF) [file pone.0190779.s007.tif]

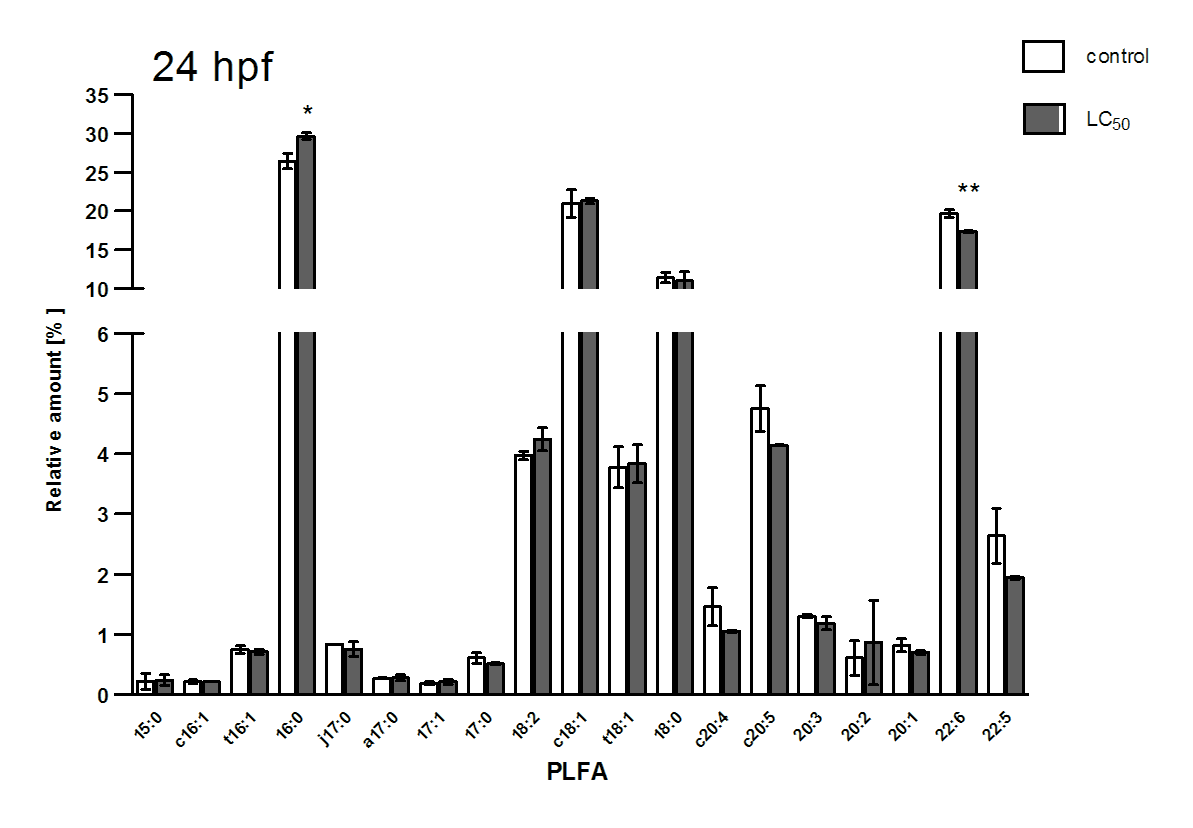

Supplement: S6 Fig — hpf: hours post fertilization; y-axis is divided into two sections with different scales; *: P < 0.05; **: P < 0.01. (TIF) [file pone.0190779.s008.tif]

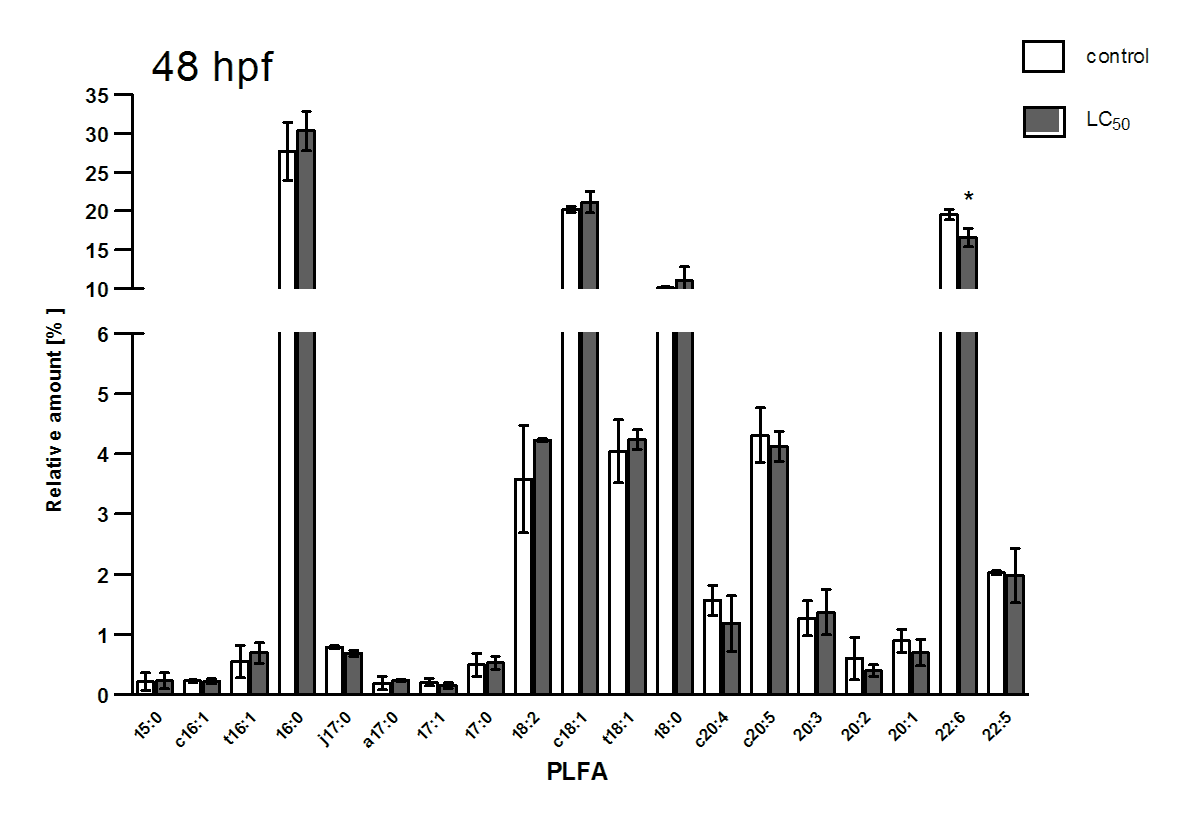

Supplement: S7 Fig — hpf: hours post fertilization; y-axis is divided into two sections with different scales; *: P < 0.05; **: P < 0.01. (TIF) [file pone.0190779.s009.tif]

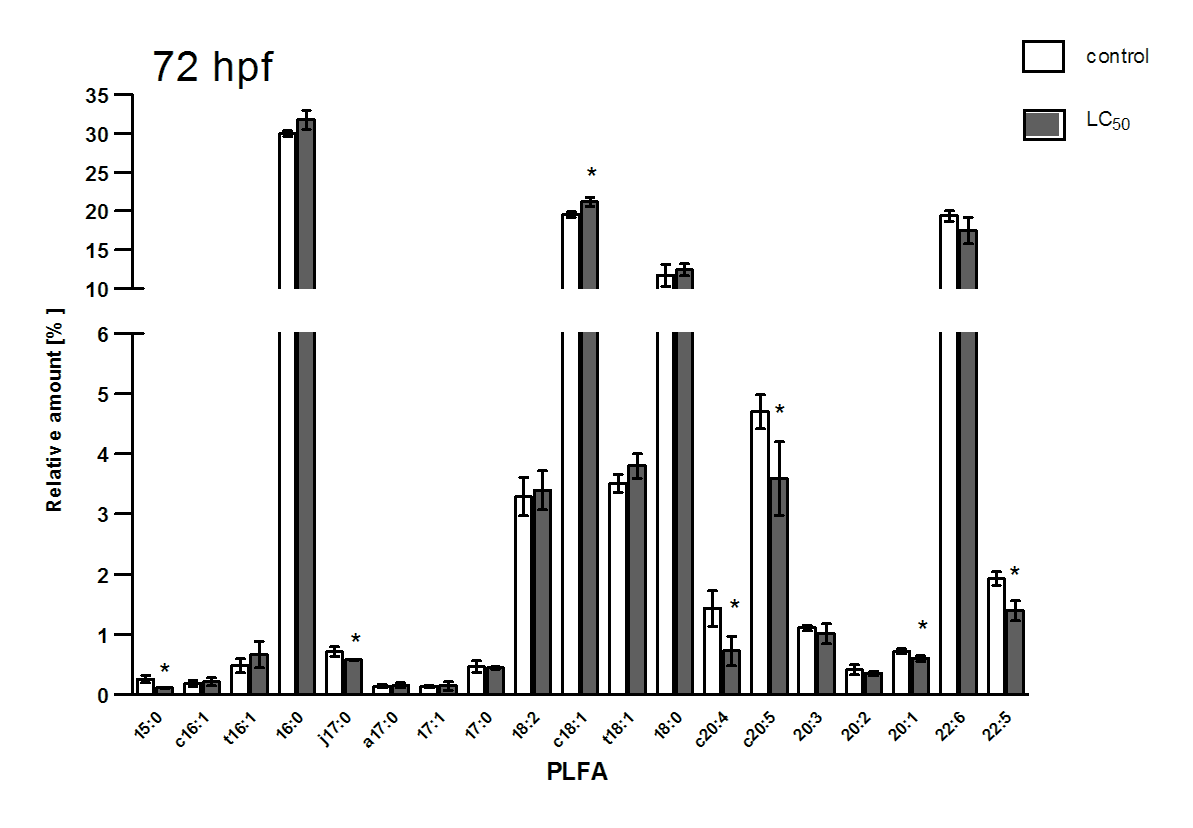

Supplement: S8 Fig — hpf: hours post fertilization; y-axis is divided into two sections with different scales; *: P < 0.05; **: P < 0.01. (TIF) [file pone.0190779.s010.tif]

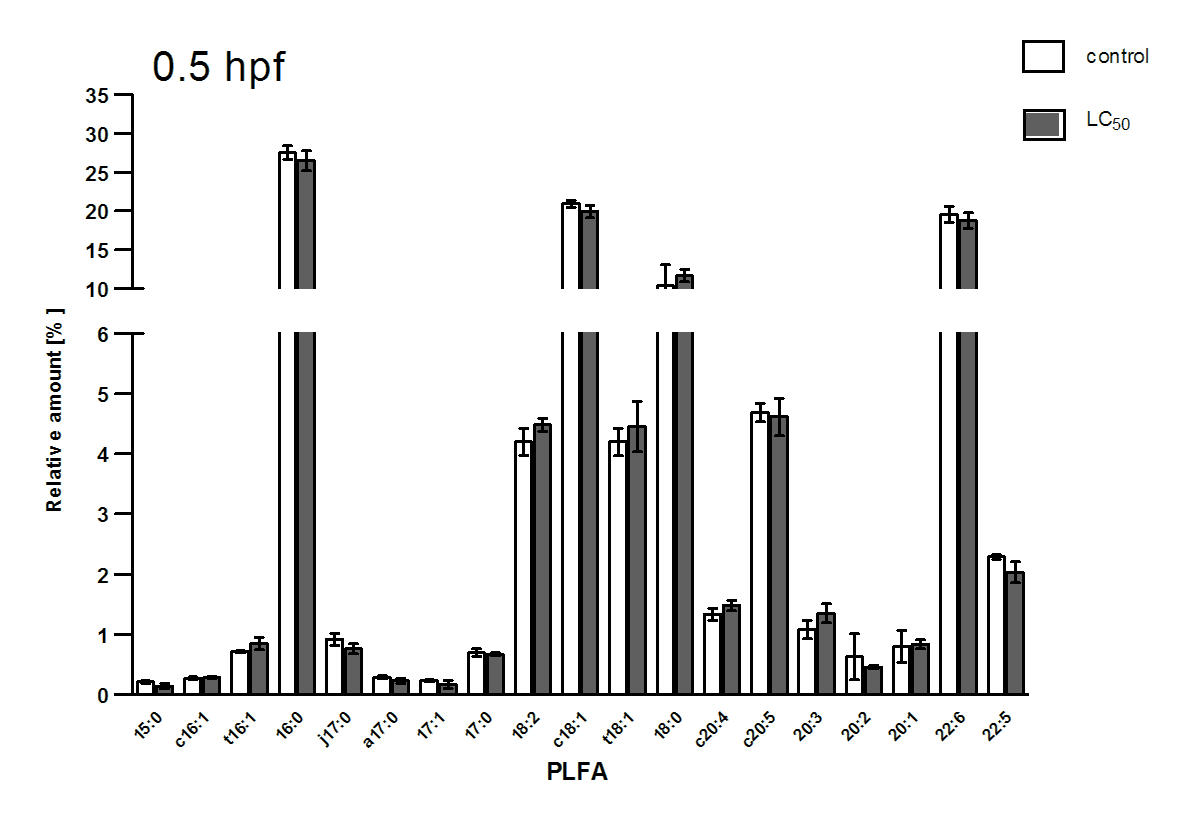

Supplement: S9 Fig — hpf: hours post fertilization; y-axis is divided into two sections with different scales; *: P < 0.05; **: P < 0.01. (TIF) [file pone.0190779.s011.tif]

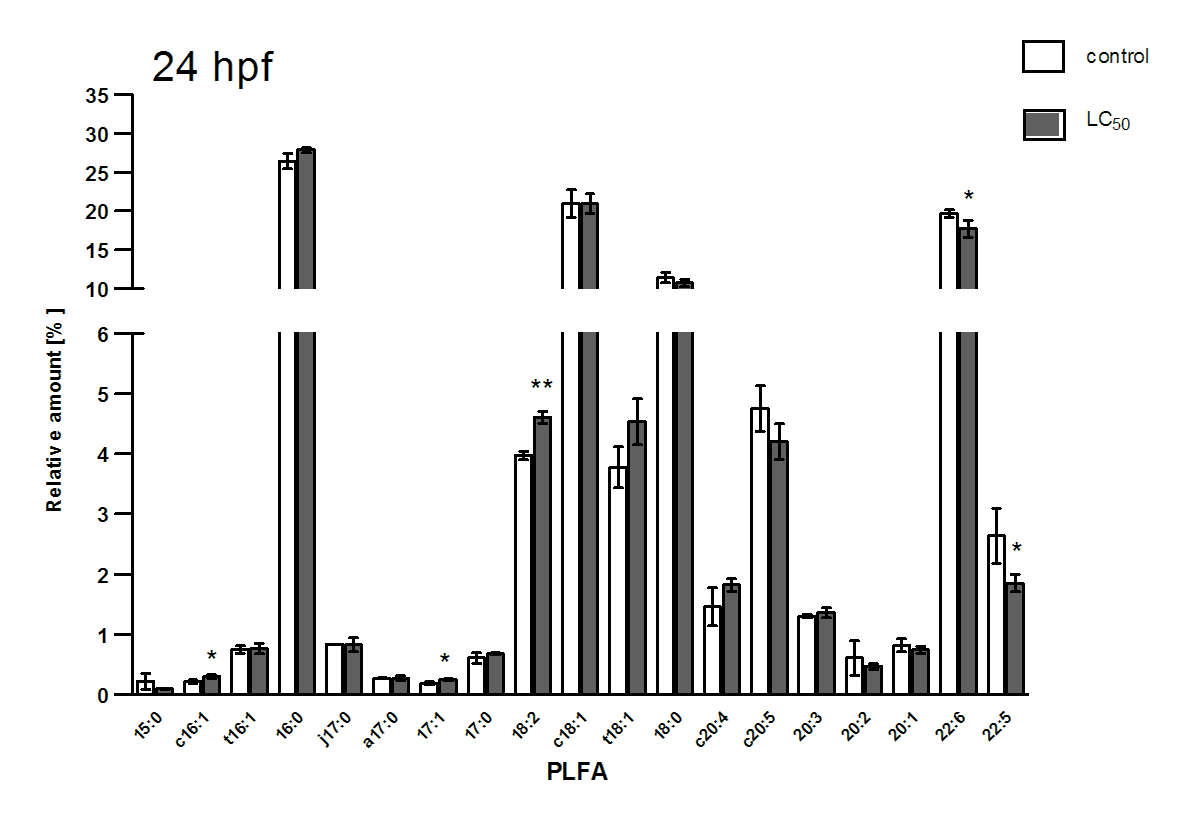

Supplement: S10 Fig — hpf: hours post fertilization; y-axis is divided into two sections with different scales; *: P < 0.05; **: P < 0.01. (TIF) [file pone.0190779.s012.tif]

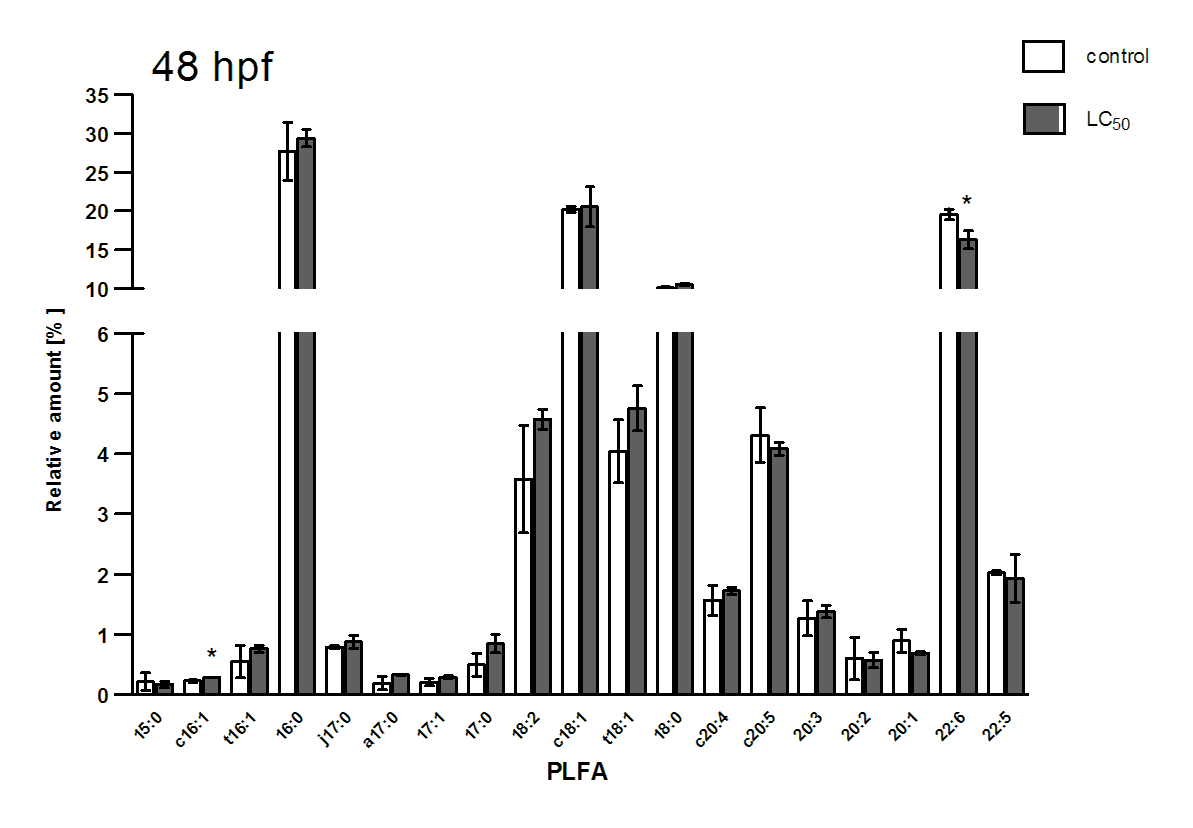

Supplement: S11 Fig — hpf: hours post fertilization; y-axis is divided into two sections with different scales; *: P < 0.05; **: P < 0.01. (TIF) [file pone.0190779.s013.tif]

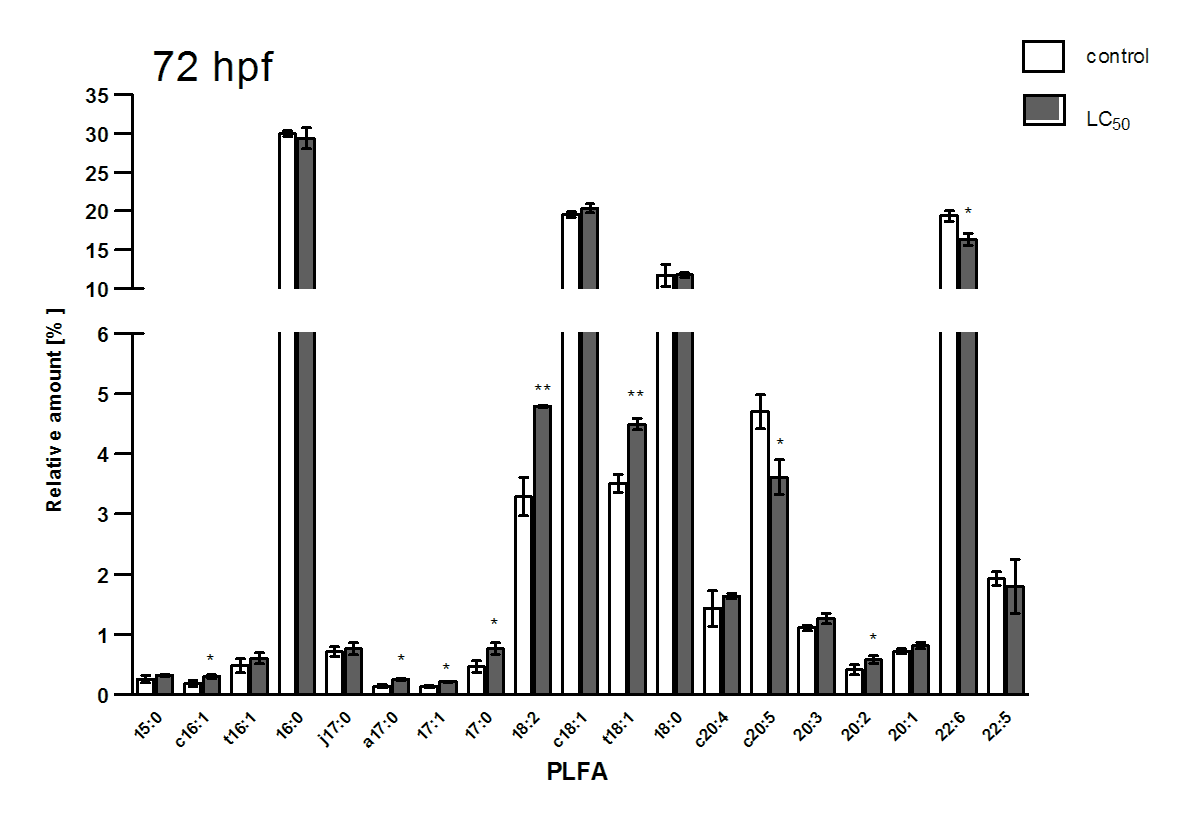

Supplement: S12 Fig — hpf: hours post fertilization; y-axis is divided into two sections with different scales; *: P < 0.05; **: P < 0.01. (TIF) [file pone.0190779.s014.tif]
